# Implications for the impairment of the rapid channel closing of *Proteomonas sulcata* anion channelrhodopsin 1 at high Cl^−^ concentrations

**DOI:** 10.1038/s41598-018-31742-6

**Published:** 2018-09-07

**Authors:** Takashi Tsukamoto, Chihiro Kikuchi, Hiromu Suzuki, Tomoyasu Aizawa, Takashi Kikukawa, Makoto Demura

**Affiliations:** 1 Faculty of Advanced Life Science, Hokkaido University, Sapporo, 060-0810 Japan; 2 Global Station for Soft Matter, Global Institution for Collaborative Research and Education, Hokkaido University, Sapporo, 001-0021 Japan; 3 Division of Macromolecular Functions, Department of Biological Sciences, School of Science, Hokkaido University, Sapporo, 060-0810 Japan; 4 Graduate School of Life Science, Hokkaido University, Sapporo, 060-0810 Japan

## Abstract

Natural anion channelrhodopsins (ACRs) have recently received increased attention because of their effectiveness in optogenetic manipulation for neuronal silencing. In this study, we focused on *Proteomonas sulcata* ACR1 (*Psu*ACR1), which has rapid channel closing kinetics and a rapid recovery to the initial state of its anion channel function that is useful for rapid optogenetic control. To reveal the anion concentration dependency of the channel function, we investigated the photochemical properties of *Psu*ACR1 using spectroscopic techniques. Recombinant *Psu*ACR1 exhibited a Cl^−^ dependent spectral red-shift from 531 nm at 0.1 mM to 535 nm at 1000 mM, suggesting that it binds Cl^−^ in the initial state with a *K*_*d*_ of 5.5 mM. Flash-photolysis experiments revealed that the photocycle was significantly changed at high Cl^−^ concentrations, which led not only to suppression of the accumulation of the M-intermediate involved in the Cl^−^ non-conducting state but also to a drastic change in the equilibrium state of the other photo-intermediates. Because of this, the Cl^−^ conducting state is protracted by one order of magnitude, which implies an impairment of the rapid channel closing of *Psu*ACR1 in the presence of high concentrations of Cl^−^.

## Introduction

Microbial rhodopsins are photoreceptor proteins produced in diverse microbes, such as archaea, bacteria and eukaryotes. The molecular functions of microbial rhodopsins are also diverse, such as light-activated ion transporters (pumps and channels) and sensors. Despite such diversities, they commonly consist of a protein moiety having 7 α-helices spanning cell membranes and a chromophore (all-*trans*-retinal) that is covalently attached to a conserved Lys residue in the 7^th^ α-helix through a protonated Schiff base linkage^1,2^. The chromophore is isomerized from an all-*trans* to a 13-*cis* configuration upon light irradiation in the time range of a few hundreds of femtoseconds, which induces sequential structural changes of the protein moiety in the time range of picoseconds to seconds. During those structural changes, several photo-intermediates are formed and then decay over time. Finally, the protein returns to its initial state. Therefore, such a light-induced reaction is cyclic and is called a photocycle. As a result, microbial rhodopsins exert individual functions during the photocycle^2^.

One type of natural ion channel rhodopsins has recently become an intensive research target because of their remarkable effectiveness in optogenetic manipulation for neuronal silencing. Those proteins are called anion channelrhodopsins (ACRs), which passively transport monovalent anions, such as halide ions and NO_3_^− ^^3,4^. So far, three kinds of ACRs have been mainly investigated. ACRs from a marine cryptophyte alga *Guillardia theta* (abbreviated as *Gt*ACR1 and 2) are the first natural ACRs reported in 2015^3^. Several *in vivo* and *in vitro* investigations have revealed their optogenetic availability^3^, their channel gating mechanism during the photocycle^5–7^, the roles of positively charged residues for anion conductance^8^, and their structure and structural changes around the chromophore^9,10^. On the other hand, another homologous ACR from a marine cryptophyte alga *Proteomonas sulcata* (abbreviated as *Psu*ACR1 or *Ps*ACR1) has also been investigated regarding its electrophysiological^4,11^ and spectroscopic properties^12^. Those studies have shown that *Gt*ACRs have the ability to work under weak light intensity and that *Psu*ACR1 has rapid channel closing kinetics, rapid dark recovery of the peak photocurrent, and the most red-shifted absorption wavelength among the known ACRs. Those characteristics are beneficial for highly sensitive, precise and rapid optogenetic manipulations. Recently, other ACRs, named ZipACR and RapACR, have been reported to be more rapid than *Psu*ACR1 and used for the optogenetics^13–15^.

Previous investigations of the channel gating mechanism of *Gt*ACR1 and *Psu*ACR1 have revealed the relationships between the photo-intermediates in the photocycle and the open and closed states of the channel^4–6,11,12^. In these cases, the anion transport starts together with the formation of the L-intermediate, which is observed in the early stage of the photocycle, whereas it stops together with the formation of the M-intermediate. These relationships in ACRs are different from those in cation channelrhodopsins^16,17^.

Focusing on the channel functions of ACRs in which several photo-intermediates are involved, anion concentration dependency, which is a useful parameter to characterize anion transport function, is still unclear. In anion pumping rhodopsins, such as archaeal and cyanobacterial halorhodopsins (HRs) and marine bacterial Cl^−^ pumping rhodopsins (ClRs), their anion transport mechanisms, including their anion binding ability, photocycle kinetics, sequence and timing of anion uptake and release, and residues important for anion transport, have been revealed based on the anion concentration dependency in their spectroscopic properties^18–26^. Therefore, in this study we used static and time-resolved absorption spectroscopy to characterize the anion channel function of ACR with varying anion concentrations, especially for Cl^−^. We focused on *Psu*ACR1 since it has rapid channel closing kinetics as described above, however the detailed mechanism involved is still unknown. *Psu*ACR1 was expressed in and extracted from methylotrophic yeast *Pichia pastoris* cells as a recombinant protein in the presence of the detergent dodecyl-β-D-maltoside (DDM). These spectroscopic measurements revealed information about the Cl^−^ binding ability in the initial state and the Cl^−^ concentration-dependent changes of the photocycle that are directly connected to its anion channel function.

## Results

### Cl^−^ dependent absorption changes in the initial state

**Figure 1:**
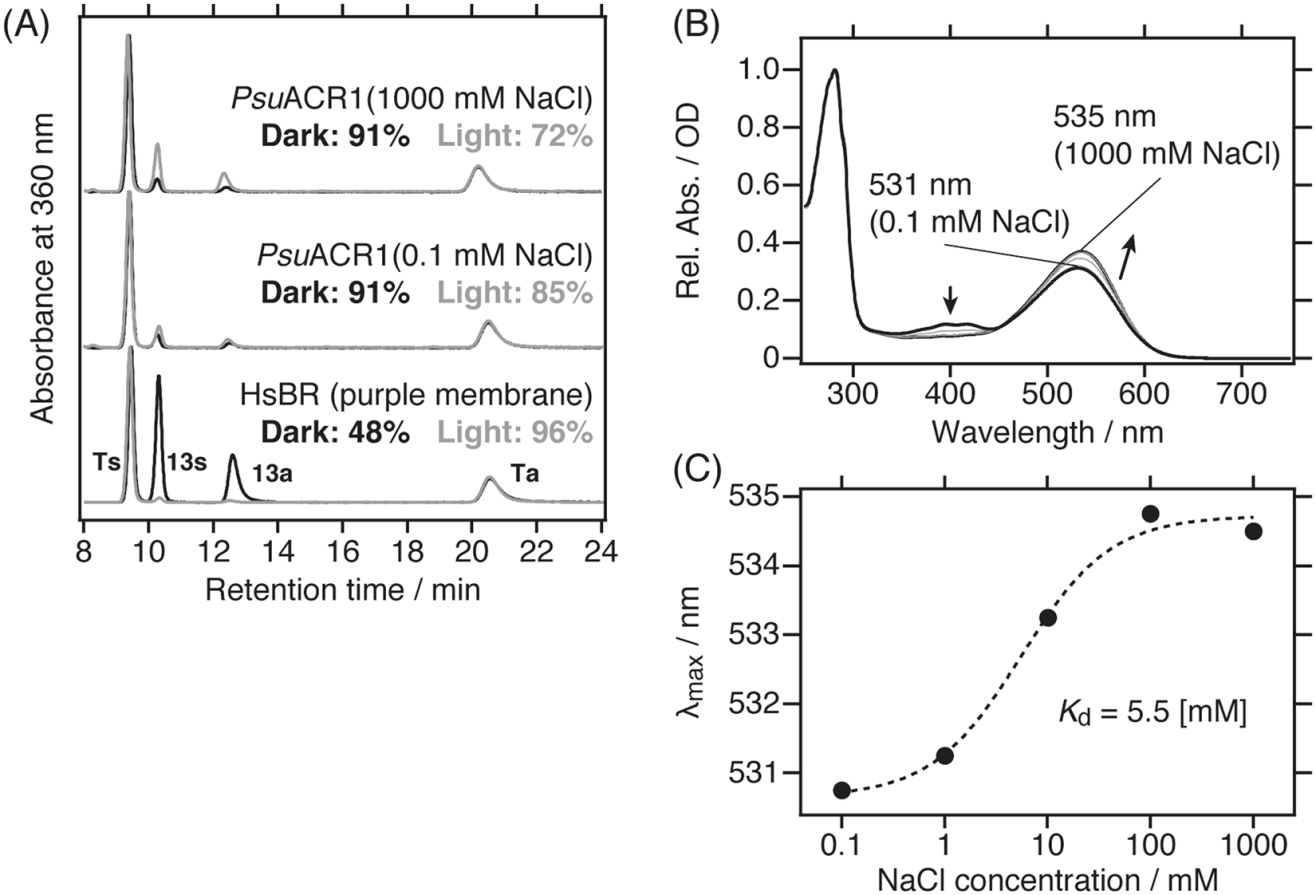
Photochemical properties of *Psu*ACR1 in the initial state. (**A**) HPLC chromatograms of retinal oxime isomers extracted under dark (black lines) and under light (grey lines) conditions in the presence of 0.1 mM or 1,000 mM Cl^−^. The 4 peaks of the retinal oxime isomers were assigned as Ts (all-*trans*, 15-*syn*), Ta (all-*trans*, 15-*anti*), 13 s (13-*cis*, 15-*syn*) and 13a (13-*cis*, 15-*anti*). The molar content of all-*trans* retinal is described in the same panel. (**B**) Static UV-visible absorption spectra of *Psu*ACR1 in the presence of 0.1, 1, 10, 100 or 1,000 mM NaCl. The directions of the Cl^−^ dependent spectral changes are indicated by arrows. (**C**) Hill plot of λ_max_ against the logarithm of Cl^−^ concentration (closed circles). The fitting curve is shown as a broken line. The fitting parameters for the Hill equation were as follows; *A* = 531 ± 0.207, *B* = 4.07 ± 0.250, *K*_d_ = 5.49 ± 1.62 and *n* = 1 (fixed).

Retinal isomer composition analysis of *Psu*ACR1 was performed using high performance liquid chromatography (abbreviated as HPLC). Figure 1A shows HPLC chromatograms of retinal oximes extracted from *Psu*ACR1 under dark or light conditions. *Psu*ACR1 showed slight dark and light adapted changes in its retinal isomer composition. With respect to the Cl^−^ concentration dependency on the retinal isomer composition, that dependency seemed to be larger in the presence of 1,000 mM Cl^−^ than in the presence of 0.1 mM Cl^−^, especially under the light condition. In summary, the chromophore composition in the initial state of *Psu*ACR1 was predominantly the all-*trans* form, which facilitates the light-gated anion channel function, at more than 90% and 70% under dark and light conditions, respectively.

To characterize the effect of Cl^−^ on the absorption properties of *Psu*ACR1 in the initial state, we measured static UV-visible absorption spectra in varying Cl^−^ concentrations from 0.1 to 1,000 mM. As shown in Fig. 1B, the visible absorption maximum (abbreviated as λ_max_) in the presence of 0.1 mM Cl^−^ was 531 nm. When the Cl^−^ concentration increased, the λ_max_ was red-shifted to 535 nm. At the same time, a minor absorption band at around 400 nm, a marker band for the deprotonated retinal Schiff base, disappeared. These results indicate that *Psu*ACR1 binds Cl^−^ in the initial state and that the bound Cl^−^ increases the acid dissociation constant (p*K*_a_) of the protonated retinal Schiff base. The same behavior was also observed for HRs^26,27^. We then estimated the Cl^−^ binding affinity of *Psu*ACR1 (the dissociation constant, *K*_d_) from the Cl^−^ dependent shift of the λ_max_. Figure 1C shows the Hill plot of λ_max_ against the Cl^−^ concentration, and from the Hill equation, the *K*_d_ was estimated to be 5.5 ± 1.6 mM.

### Photocycle of *Psu*ACR1 in the presence of 100 mM Cl^−^

**Figure 2:**
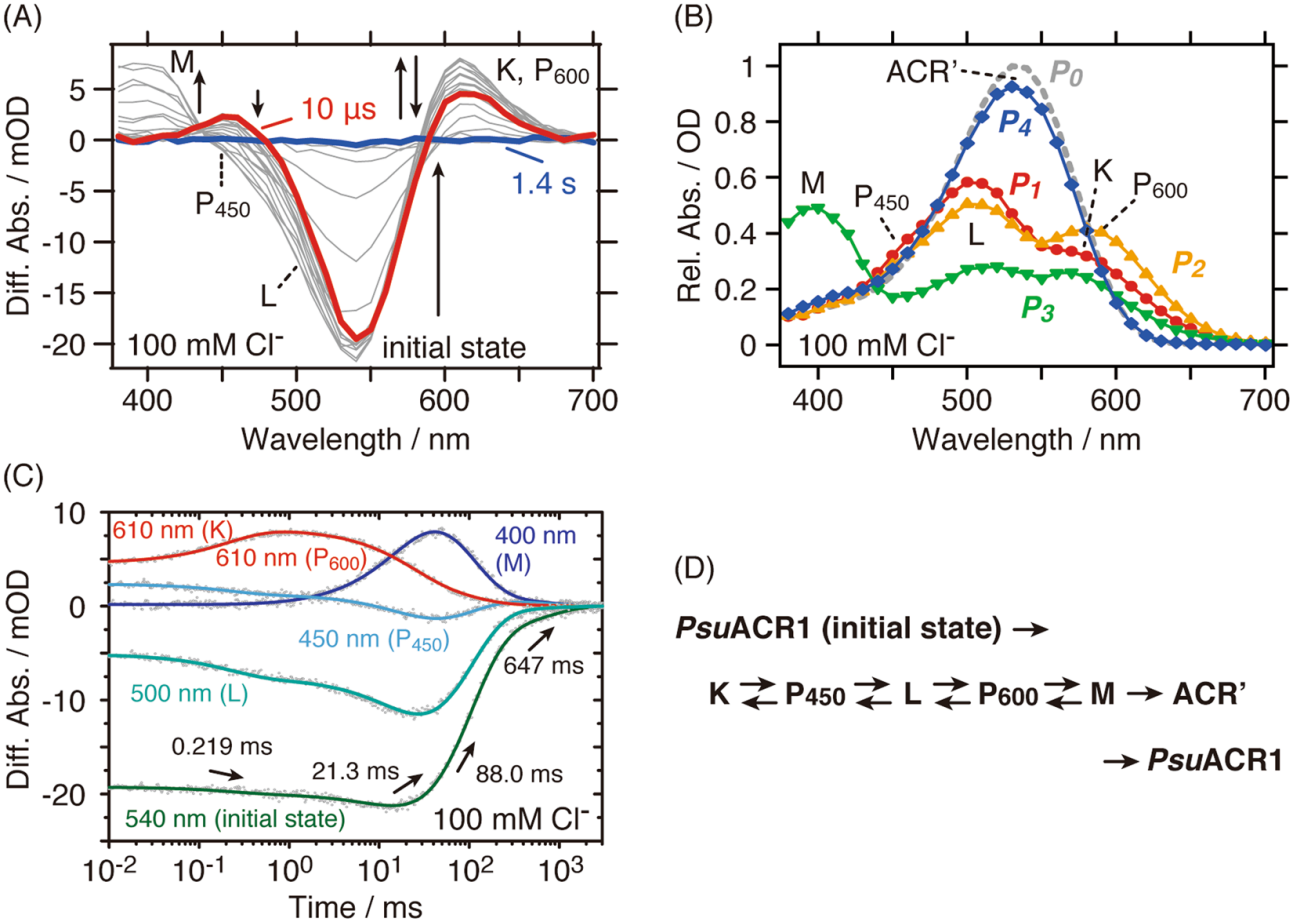
Photocycle kinetics of *Psu*ACR1 in the presence of 100 mM Cl^−^. (**A**) Flash-induced light minus dark difference absorption spectra. Red and blue lines represent the spectra at 10 μs and 1.4 s, respectively. The directions of the evolution of the spectral changes are indicated by arrows. (**B**) Absorption spectra of the *P*_1_–*P*_4_ states (red circles, orange triangles, green inverted triangles and blue diamonds, respectively) calculated from the fitting results. The spectrum of *P*_0_ (grey broken line) represents the pure retinal spectrum. (**C**) Time evolution of flash-induced absorption changes (shown as grey dots) of the initial state (540 nm), K and P_600_ (610 nm), L (500 nm), P_450_ (450 nm) and M (400 nm), respectively. Fitting curves are shown as solid lines with colors. Time constants are shown in the same panel. (**D**) Photocycle scheme in the presence of 100 mM Cl^−^.

The photocycle of *Psu*ACR1 was investigated using time-resolved flash-photolysis in the time range of microseconds to seconds, during which the protein exerts its Cl^−^ channel function. Here we explain the photocycle overview in the presence of 100 mM Cl^−^ as an example. Figure 2A illustrates the flash-induced light-minus-dark difference absorption spectra from 10 μs to 1.4 s. After the flash excitation, the absorption for the initial state at 540 nm disappeared together with the concomitant appearance of three photo-intermediates with absorptions at 610 nm, 450 nm and 400 nm, tentatively assigned as K-, P_450_- and M-intermediates (abbreviated as K, P_450_ and M), respectively^6,12^. Over time, these photo-intermediates increased and then decreased together with the recovery of the initial state and therefore the photocycle was completed.

To examine the photocycle kinetics precisely, we performed global fitting analysis based on the sequential model^28,29^. These data were successfully fitted by the exponential decay functions with the sum of 4 exponents, indicating that at least 4 kinetically defined states, *P*_1_–*P*_4_, were detected in our experimental time domain (Supplementary Fig. S1). Figure 2B shows the absorption spectra for the *P*_1_–*P*_4_ states calculated using the fitting results. *P*_0_ represents the pure retinal spectrum of the initial state *Psu*ACR1. From the spectra, we assigned two additional photo-intermediates with absorption peaks at 500 nm and 540 nm as the L- and ACR’-intermediates (abbreviated as L and ACR’), respectively, by reference to previous reports^6,12,23–25^.

Figure 2C shows the time-dependent absorption changes of representative photo-intermediates as described above. Due to the time resolution of our flash-photolysis apparatus (10 μs), which is larger than that of a previous report by two orders of magnitude^11^, the observed photocycle started from the equilibrium state between K (610 nm) and L (500 nm). The *P*_1_ spectrum shown in Fig. 2B supports their equilibrium. In addition, the spectral shoulder corresponding to P_450_ (450 nm) was observed in the *P*_1_ state (Fig. 2A,B), indicating the co-existence with K and L. The *P*_1_ state decayed to the *P*_2_ state at the time constant τ_1_ (0.219 ms, Fig. 2C). During this transition, the absorption at 610 nm transiently increased together with the decrease in the absorption at 500 nm. In a previous study, these transient absorption changes corresponded to the re-establishment of the K/L equilibrium in favor of K^12^. However, based on the conventional photocycle scheme, it is more straight-forward to assign the transient increase in 610 nm as the generation of a new photo-intermediate rather than the re-establishment of the K/L equilibrium. Therefore, we adopted the P_600_-intermediate (abbreviated as P_600_), whose λ_max_ was estimated to be 600 nm from the *P*_2_ spectrum in Fig. 2B, as an intermediate followed by L. Previous reports for *Psu*ACR1 indicated the existence of an intermediate similar to P_600_ named P_620_^11^ or K_2_^12^. In summary, an equilibrium state among P_450_, L and P_600_ was observed in the *P*_2_ state (Fig. 2B). The *P*_2_ state decayed to the *P*_3_ state at the time constant τ_2_ (21.3 ms, Fig. 2C). The *P*_3_ state in the presence of 100 mM Cl^−^ contained L, P_600_, and M (400 nm) at the same time (Fig. 2B). The *P*_3_ state was then converted to the *P*_4_ state at the time constant τ_3_ (88.0 ms, Fig. 2C), where ACR’ (540 nm) mainly populated (Fig. 2B). Finally, the *P*_4_ state decayed to the initial state *P*_0_ at the time constant τ_4_ (647 ms, Fig. 2C) to close the photocycle. From the analysis described here, we summarize the photocycle scheme of *Psu*ACR1 in the presence of 100 mM Cl^−^ in Fig. 2D.

### Cl^−^ dependence on the photocycle

**Figure 3:**
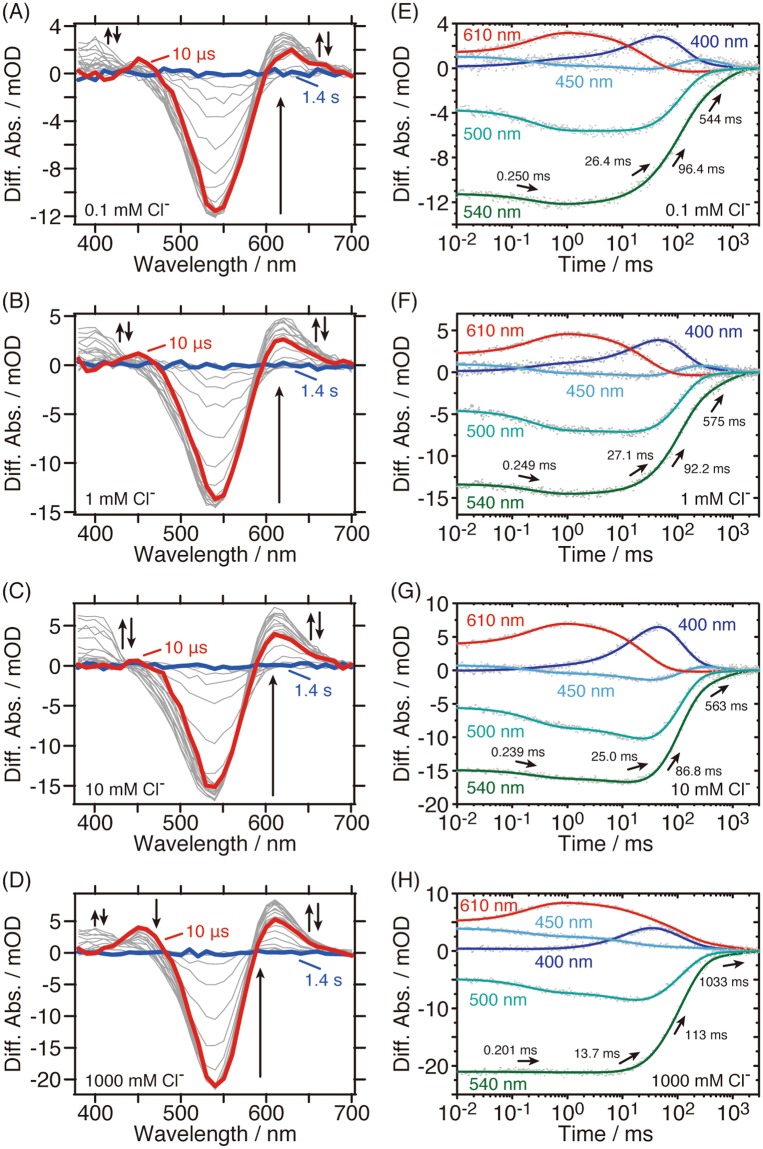
The Cl^−^ dependence on the photocycle kinetics of *Psu*ACR1 in the presence of (**A**,**E**) 0.1, (**B**,**F**) 1, (**C**,**G**) 10 and (**D**,**H**) 1,000 mM Cl^−^. (Left panels) Flash-induced light minus dark difference absorption spectra. Red and blue lines represent the spectra at 10 μs and 1.4 s, respectively. The directions of the evolution of the spectral changes are indicated by arrows. (Right panels) Time evolution of flash-induced absorption changes (shown as grey dots) of the initial state (540 nm), K and P_600_ (610 nm), L (500 nm), P_450_ (450 nm) and M (400 nm), respectively. Fitting curves are shown as solid lines with colors. Time constants are shown in the same panel.

The Cl^−^ dependence on the photocycle of *Psu*ACR1 was also investigated by flash-photolysis. Figure 3 illustrates the light-minus-dark difference absorption spectra and the time dependent absorption changes of the representative photo-intermediates in the presence of 0.1–1,000 mM Cl^−^, except for 100 mM Cl^−^. From these results, two major effects of the Cl^−^ concentration on the photocycle were identified: (i) The accumulations of P_450_ and M changed with increases in the Cl^−^ concentration (Figs 2A and 3A–D); and (ii) The lifetime of P_600_ was prolonged and therefore its decay was synchronized with that of M in the presence of more than 1,000 mM Cl^−^ (Fig. 3H). The same effects were observed in the presence of 4,000 mM Cl^−^ (Supplementary Fig. S2).

**Figure 4:**
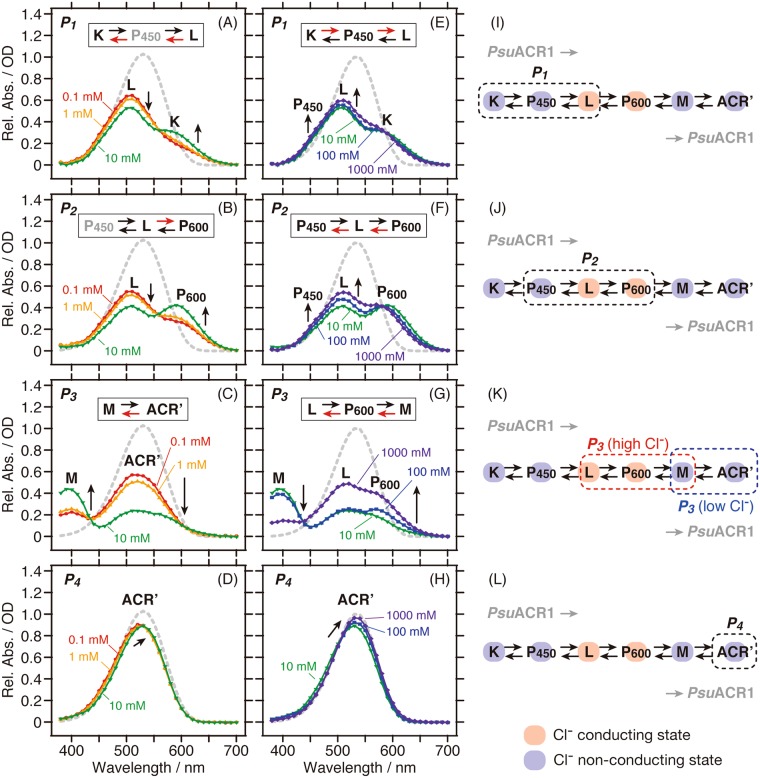
The Cl^−^ dependence on the absorption spectra of the *P*_1_–*P*_4_ states (from the top to the bottom) in the presence of (**A**–**D**) 0.1–10 mM (red, orange and green, respectively) and (**E**–**H**) 10–1,000 mM Cl^−^ (green, blue and purple, respectively). The spectrum of *P*_0_ (grey broken line) represents pure retinal spectra. The arrows indicate the direction of the changes in absorption and accumulation. For better understanding, the assignment and the equilibrium state of the photo-intermediates are shown in each panel. The Cl^−^ dependent equilibrium shift is indicated by the red arrow. (**I**–**L**) The photo-intermediates in the *P*_1_–*P*_4_ states are illustrated in the photocycle scheme of *Psu*ACR1. The Cl^−^ conducting and non-conducting states are highlighted in red and blue, respectively.

To analyze the Cl^−^ dependence on the photocycle kinetics in detail, we compared the absorption spectra of the *P*_1_–*P*_4_ states at each Cl^−^ concentration (Fig. 4). From that analysis, we found that the Cl^−^ dependence changed at 10 mM Cl^−^. Therefore, we separately prepared the absorption spectra in the presence of 0.1–10 mM (panels A–D) and 10–1,000 mM (panels E–H). In the *P*_1_ state, where K, P_450_ and L were in equilibrium (Fig. 4A,E and I), the equilibrium shifted from L to K in the presence of 0.1–10 mM, whereas slight increases in L and P_450_ were observed in the presence of 10–1,000 mM. Similarly, in the *P*_2_ state, where P_450_, L and P_600_ were in equilibrium (Fig. 4B,F and J), the equilibrium shifted from L to P_600_ in the presence of 0.1–10 mM Cl^−^, while increases in L and P_450_ were observed in the presence of 10–1,000 mM Cl^−^. The spectra were significantly changed in the *P*_3_ state (Fig. 4C,G and K). In the presence of 0.1–10 mM Cl^−^ (Fig. 4C,K), M and ACR’ accumulated and the equilibrium shifted from ACR’ to M. In addition, the spectrum in the presence of 10 mM Cl^−^ seemed to contain L and P_600_ other than ACR’ at the same time (Fig. 4C,G), indicating there is a transition phase between the photocycle in the presence of lower or higher concentrations of Cl^−^. When increasing the Cl^−^ concentration from 10 to 1,000 mM (Fig. 4G,K), L and P_600_ were clearly observed in the spectra. These intermediates were in equilibrium with M and the equilibrium shifted from M to L and P_600_. Therefore, such a Cl^−^ dependent equilibrium shift resulted in an increase and a decrease in the accumulation of M and the prolongation of the lifetime of P_600_, respectively, which was also supported by the results shown in Fig. 3H. In the *P*_4_ state, where ACR’ mainly accumulated, absorption changes of ACR’ were detected (Fig. 4D,H and L), which reflects that Cl^−^ was taken up during the lifetime of ACR’.

## Discussion

In this study, we investigated the Cl^−^ dependent changes in the photochemical properties of *Psu*ACR1 using static and time-resolved spectroscopic techniques that revealed that the photocycle, which is directly connected to the anion channel function, is strongly affected by Cl^−^ concentration.

### Indication for Cl^−^ binding in the initial state

We demonstrated that the visible absorption of *Psu*ACR1 shifted with changes in the Cl^−^ concentration (Fig. 1B). In a previous study of *Psu*ACR1, the λ_max_ in the absence or presence of Cl^−^ both resulted in 534 nm, which is close to our results in the presence of more than 100 mM Cl^−^ (Fig. 1C), and thus no spectral shift was observed^12^. Currently, we cannot clearly explain why such a difference occurred. One possible reason may be that in the previous study more than a certain concentration (e.g. the *K*_d_ of 5.5 mM determined in this study) of Cl^−^ remained in the sample solution even after the buffer exchange. Incidentally, in the case of a homologous protein *Gt*ACR1, no spectral shift was observed between 0 and 300 mM Cl^− ^^6^. The difference in the initial state Cl^−^ binding between *Psu*ACR1 and *Gt*ACR1 will be an interesting issue.

*Psu*ACR1 showed a Cl^−^ induced spectral red-shift (Fig. 1B), which is opposite to the case of many Cl^−^ pumping rhodopsins, such as the haloarchaeal *Natronomonas pharaonis* HR (NpHR)^18,22,23^ and the bacterial *Nonlabens marinus* S1-08^T^ rhodopsin 3 (NM-R3)^25^. On the other hand, spectral red-shifts similar to those of *Psu*ACR1 were observed in haloarchaeal *Halobacterium salinarum* HR (HsHR)^20^, in bacterial *Mastigocladopsis repens* HR (MrHR)^30^ and in *Salinibacter ruber* sensory rhodopsin I (SrSRI)^31^. For the latter red-shifted species, two different Cl^−^ binding sites are hypothesized. One is in the vicinity of the protonated retinal Schiff base, which was revealed by crystal structure and spectroscopic measurements in HsHR^32^ and MrHR^30^. To confirm this, we prepared a mutant of *Psu*ACR1 for Ala93, which corresponds to Thr74 in MrHR, Thr126 in NpHR, and Asp85 in HsBR (Supplementary Fig. S3A). In the cases of MrHR and NpHR, amino acid substitutions at the 74^th^ and 126^th^ positions from Thr to acidic residues resulted in the disappearance of the spectral shift and thus the initial Cl^−^ binding ability^30,33^. Therefore, we prepared the *Psu*ACR1-A93E mutant with the hope of the same results obtained for the MrHR and NpHR mutants. Supplementary Fig. S3B shows the absorption spectra of *Psu*ACR1-A93E in the presence of 0.1 mM or 1,000 mM Cl^−^. Unexpectedly, a Cl^−^ dependent spectral red-shift from 504 nm (0.1 mM) to 508 nm (1,000 mM) was observed. Therefore, it is unlikely that *Psu*ACR1 shares the same Cl^−^ binding site with MrHR and NpHR in the initial state.

The other hypothesis about the initial Cl^−^ binding site is that it resides in the vicinity of the β-ionone ring of the retinal chromophore, which has been reported in SrSRI^31^. In this case, His131 near the β-ionone ring is involved in the Cl^−^ binding, where the bound Cl^−^ induces the delocalization of the positive charge on the protonated retinal Schiff base nitrogen towards the β-ionone ring that induces the spectral red-shift. To confirm this for *Psu*ACR1, we found that His131 in SrSRI was substituted to Phe156 in *Psu*ACR1 (Supplementary Fig. S3A). We further searched for other candidates having a positive charge, however such residues were not found near the β-ionone ring of the retinal in *Psu*ACR1. Therefore, we successfully demonstrated the Cl^−^ binding ability of *Psu*ACR1 in the initial state but identification of the specific binding site must await future study.

With regard to the Cl^−^ binding affinity, the *K*_d_ was estimated to be 5.5 ± 1.6 mM from the Hill equation (Fig. 1C), which was in the same order as HsHR (2.6 mM)^34^, NpHR (5.0 mM)^35^, bacterial *Rubricoccus marinus* HR (RmHR; 7.6 mM)^26^ and MrHR (2.0 mM)^30^. This result indicates that the natively expressed *Psu*ACR1 in *P. sulcata* binds Cl^−^ in the initial state under physiological conditions (the Cl^−^ concentration in the marine environment is a few hundreds of millimolar).

### Effects of Cl^−^ concentration on the channel function of *Psu*ACR1: Relationships between photo-intermediates and Cl^−^ conducting and non-conducting states

Previous reports for *Gt*ACR1 and *Psu*ACR1 described the photo-intermediates in the photocycle as corresponding to the anion-conducting and non-conducting states by combining spectroscopic and electrophysiological results^4–6,11,12^. In those cases, the anion conductance starts and stops when forming the L and M photo-intermediates, respectively. Therefore, L and the following P_600_ in our photocycle model are involved in the Cl^−^ conducting state and K, P_450_, M and ACR’ are involved in the Cl^−^ non-conducting state (Fig. 4I–L).

**Figure 5:**
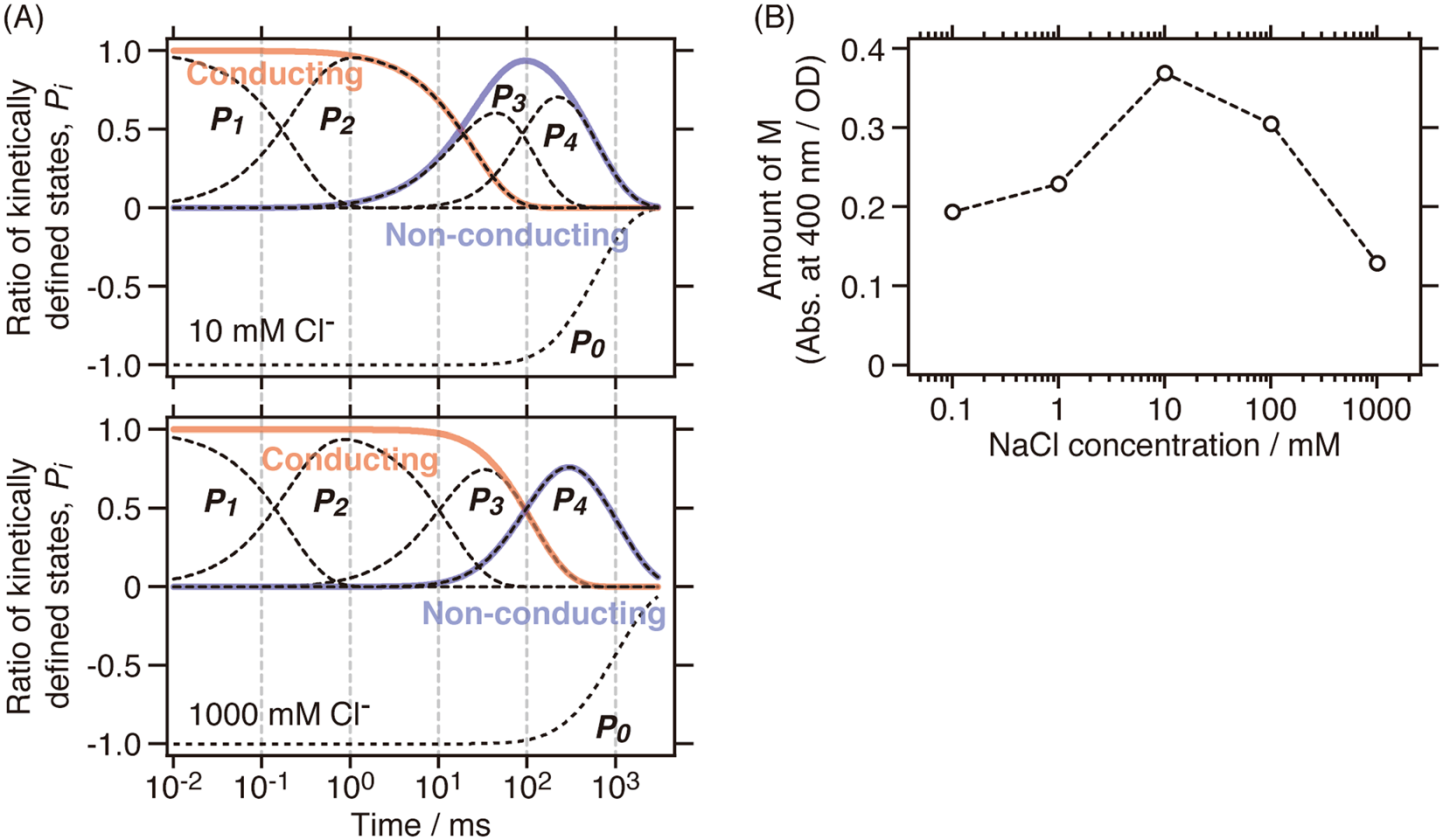
Cl^−^ concentration dependence of the photo-intermediates. (**A**) Time evolution of the kinetically defined states *P*_1_–*P*_4_ in the presence of lower (e.g. 10 mM, upper panel) and higher (e.g. 1,000 mM, lower panel) concentrations of Cl^−^. Cl^−^ conducting and non-conducting states of *Psu*ACR1 are shown as red and blue lines, respectively. (**B**) The amount of M in the *P*_3_ state (black) is plotted against the logarithm of the Cl^−^ concentration. The amount was calculated by subtracting the absorbance at 400 nm in the initial *P*_0_ state from that in the *P*_3_ state for M at each Cl^−^ concentration.

We clearly observed a Cl^−^ dependent change of the photocycle kinetics, especially in the equilibrium states of the photo-intermediates in the presence of higher concentrations of Cl^−^ (10–1,000 mM, Figs 3 and 4). Notably, we identified the most drastic change in the *P*_3_ spectra in the presence of a higher Cl^−^ concentration (Fig. 4C,G). The *P*_3_ state in the presence of 100–1,000 mM Cl^−^ is considered to be the Cl^−^ conducting state due to the significant equilibrium shift from M to L and P_600_ (Fig. 4G,K), whereas that in the presence of 0.1–1 mM Cl^−^ is considered to be the Cl^−^ non-conducting state due to the co-existence of M and *Psu*AR1’ in equilibrium (Fig. 4C,K). The *P*_3_ spectrum in the presence of 10 mM Cl^−^ corresponds to the mixture of the Cl^−^ conducting and non-conducting states. Based on the relationships between the photo-intermediates and the Cl^−^ conducting and non-conducting states as described above, the *P*_1_ and *P*_2_ states correspond to the Cl^−^ conducting state, and the *P*_3_ and *P*_4_ states correspond to the non-conducting states in the presence of lower concentrations of Cl^−^ (see also Fig. 4I–L). On the other hand, in the presence of higher concentrations of Cl^−^ (e.g. 1,000 mM), the *P*_1_–*P*_3_ states correspond to the Cl^−^ conducting state, and the *P*_4_ state corresponds to the non-conducting state (see also Fig. 4I–L). Figure 5A shows the time course for the generation and decay of the *P*_1_–*P*_4_ states. This result clearly indicates that the Cl^−^ conducting state is protracted by one order of magnitude in the presence of higher concentrations of Cl^−^. From these results, we hypothesize that one of the most pronounced characteristics of *Psu*ACR1, i.e. the rapid channel closing and rapid dark recovery of the photocurrent^4,11^, which enables the optogenetic neuronal silencing at rapid frequency, becomes impaired at higher concentrations of Cl^−^.

In addition, we noticed that the accumulation of M, which is involved in the Cl^−^ non-conducting state, changed in a Cl^−^ concentration-dependent manner, as shown in Fig. 5B. The accumulation of M first increased, then reached a maximum at 10 mM Cl^−^, and finally decreased with the increase in Cl^−^ concentration. From the Cl^−^ concentration dependency, we estimated that the Cl^−^ concentration for the first transition is close to the *K*_d_ value for the initial Cl^−^ binding (5.5 mM, Fig. 1C). On the other hand, the Cl^−^ concentration for the second transition was estimated to be several hundreds of millimolar. We suppose that in the presence of a higher Cl^−^ concentration than this value, a secondary Cl^−^ binding occurs in *Psu*ACR1 that significantly inhibits the accumulation of M. Therefore, we propose that there is a causal relationship between the secondary Cl^−^ binding and the impairment of the rapid channel closing of *Psu*ACR1 at higher concentrations of Cl^−^. Although the secondary Cl^−^ binding site has not been identified yet, we estimate that it is located near or along the Cl^−^ conducting pathway in the protein. Previously, the inhibitory role of the Arg residue on the extracellular surface of *Gt*ACR2, which is a candidate consisting of the Cl^−^ conducting pathway, for its anion channel function has been reported^8^. One of the authors’ discussion points regarding the inhibition mechanism is that the positively charged Arg84 interacts with the negatively charged Cl^−^, which prevents the Cl^−^ from being transported through the protein^8^. Together with the fact that *Psu*ACR1 conserves the corresponding residue as Arg84, we estimate that the inhibitory role of Arg84 is related to the secondary Cl^−^ binding and therefore the Arg84 in *Psu*ACR1 is one candidate for the secondary Cl^−^ binding site. If the secondary Cl^−^ binding occurs on the protein surface near Arg84, a mutation to destroy that secondary binding site would enable optogenetic silencing at a high frequency through *Psu*ACR1 even in the presence of higher Cl^−^ concentrations. Another estimation is that water-filled cavities along the channel pathway in *Psu*ACR1 contribute to the secondary Cl^−^ binding. X-ray and simulation structures of cation channelrhodopsins (CCRs) C1C2 and ChR2 from *Chlamydomonas reinhardtii* indicated that such cavities are distributed along the possible cation channel pathway and predicted to be involved in the cation permeation^36–39^. Moreover, in the case of Cl^−^ conducting mutant of C1C2 (C1C2-E90K/R), the distribution of the cavities was expanded, which facilitated Cl^−^ distributed in the cavities and resulted in the increase in the affinity for Cl^−^ ^40^. In analogy with these CCRs, there should be similar water-filled cavities in ACRs including *Psu*ACR1 to capture Cl^−^. We hypothesize that several Cl^−^ are captured by the cavity during the Cl^−^ conducting L- or P_600_-intermediate in the presence of high concentrations of Cl^−^, which may stabilize the L or P_600_ and thus remain the channel open.

The currently fastest ACR for optogenetic silencing, called ZipACR, originates from *P. sulcata* and thus is a homologous protein with *Psu*ACR1 (identity 32%, similarity 71%)^13^. On the other hand, *Gt*ACR1 also shares a high sequential homology with *Psu*ACR1 (identity 36%, similarity 74%). Based on our hypothesis, the similar Cl^−^ dependence and thus impairment of the channel closing in the presence of higher concentrations of Cl^−^ may occur in these homologous ACRs.

## Conclusion

In this study, we analyzed the Cl^−^ dependent changes in the photochemical properties of *Psu*ACR1 using static and time-resolved spectroscopic techniques. We found that *Psu*ACR1 is able to bind Cl^−^ in the initial state at a *K*_d_ of 5.5 mM, which was estimated by the Cl^−^ dependent spectral red-shift. In addition, the Cl^−^ concentration dependency on the photocycle was clearly observed. In the presence of more than 10 mM Cl^−^, the photocycle of *Psu*ACR1 was significantly changed as follows; (i) the accumulation of M, which is involved in the Cl^−^ non-conducting state, was strongly suppressed, and (ii) due to (i) and the drastic change in the equilibrium state of the other photo-intermediates, the Cl^−^ conducting state was protracted by one order of magnitude compared to that in the presence of lower concentrations of Cl^−^. These results suggest that the most pronounced characteristics of *Psu*ACR1, rapid channel closing and rapid dark recovery of the photocurrent, which enables the rapid optogenetic manipulation for neuronal silencing, becomes impaired in the presence of high concentrations of Cl^−^. We propose that there is a causal relationship between the secondary Cl^−^ binding and the impairment of the rapid channel closing of *Psu*ACR1 at high concentrations of Cl^−^. For the present use of ACRs for optogenetics, the proteins may not be exposed to such high anion concentrations condition in neurons. However, we hope that our study will be helpful to engineer optogenetic tools based on ACRs.

## Methods

### DNA construction of *Psu*ACR1

The amino acid sequence of *Psu*ACR1 was the same as previously reported (GenBank: KF992074.1, 291 residues)^4,11,12,41^. For affinity purification, 8 histidine residues were attached to the C-terminus of *Psu*ACR1 (abbreviated as *Psu*ACR1_His_8_). The gene encoding *Psu*ACR1_His_8_ with the codon optimization for *Pichia pastoris* was purchased from GENEWIZ (South Plainfield, NJ, USA). Two restriction enzyme sites, EcoRI and NotI, were attached to the 5′- and 3′-teminal ends of the *Psu*ACR1_His_8_ gene, and a stop codon was introduced before the NotI site. According to this, we obtained *Psu*ACR1_His_8_ having 299 residues in total. The gene and the expression vector pPICZ B (Thermo Fisher Scientific, Waltham, MA, USA) were digested by EcoRI and NotI restriction enzymes (Roche, Basel, Switzerland) and were then ligated using a Mighty Mix DNA ligation kit (Takara Bio Inc., Shiga, Japan). Nucleotide displacement was introduced using a QuikChange Site-Directed Mutagenesis kit (Agilent Technologies, Santa Clara, CA, USA) to produce *Psu*ACR1-A93D and *Psu*ACR1-A93E mutants. The nucleotide sequences were verified by the dideoxy sequencing method using a BigDye Terminator v1.1 Cycle Sequencing kit and a 3130 DNA Analyzer (Applied Biosystems, Foster City, CA, USA).

### Protein expression and purification

The methylotrophic yeast *Pichia pastoris* SMD1168H strain (Thermo Fisher Scientific) was used as the protein expression host. For the transformation of *P. pastoris*, pPICZ B_*Psu*ACR1_His_8_ plasmid DNA was linearized using the PmeI restriction enzyme (New England Biolabs, Ipswich, MA, USA), purified using a FastGene Gel/PCR Extraction kit (NIPPON Genetics Co., Ltd, Tokyo, Japan), and then introduced to competent *P. pastoris* cells by a standard electroporation method. The transformed *P. pastoris* cells were inoculated and pre-cultured in BMGY medium containing 100 μg/mL Zeocin^TM^ (Thermo Fisher Scientific) for two days at 30 °C. The medium was exchanged to BMMY medium containing 0.5% methanol, 100 μg/mL Zeocin^TM^ and 10 μM all-*trans*-retinal (Sigma Aldrich, St. Louis, MO, USA), and protein expression was induced for 24 hr at 30 °C. After the protein induction, the cells were collected by centrifugation, resuspended in 50 mM Tris-HCl (pH 8.0) buffer containing 300 mM NaCl, and then sufficiently disrupted at 4 °C using a French press (100 MPa, repeated 6 times; Ohtake, Tokyo, Japan). The cell suspension was centrifuged at 7,000 rpm for 5 min at 4 °C (TOMY EX-136 equipped with a TLA-11 rotor; TOMY Seiko Co., Ltd., Tokyo, Japan) and the supernatant containing membrane fraction was collected. The membrane fraction was collected by ultracentrifugation at 40,000 rpm for 1 hr at 4 °C (Hitachi Koki CP 90NX equipped with a P70AT rotor; Hitachi Koki Co., Ltd., Tokyo, Japan). The procedures for solubilization with DDM (Dojindo Laboratories, Kumamoto, Japan) and affinity purification were the same as previously reported^25^. For spectroscopic measurements, the buffer was sufficiently exchanged with 10 mM MOPS buffer (pH 7.0, Dojindo Laboratories) containing the desired concentrations of NaCl (0.1, 1, 10, 100 and 1,000 mM) and Na_2_SO_4_ (0, 300, 330, 333 and 333.3 mM) by centrifugation for 10 times (Amicon Ultra centrifuge filter, 30,000 molecular weight cut-off, Merck Millipore, Burlington, MA, USA) and gel-filtration chromatography (PD-10 column, GE Healthcare, Chicago, IL, USA). The ionic strength was kept at 1,000 mM by adding Na_2_SO_4_ because SO_4_^2−^ is impermeable for *Psu*ACR1^4^. For spectroscopic measurements at high salt concentrations, we prepared *Psu*ACR1 samples in the same MOPS buffer containing 0.05% DDM and 4,000 mM NaCl or 1,333.3 mM Na_2_SO_4_. The ionic strength was kept at 4,000 mM. The same procedures were used to produce the *Psu*ACR1-A93D and *Psu*ACR1-A93E mutants. However, the *Psu*ACR1-A93D mutant was not functionally expressed in the cells.

### Retinal isomer composition analysis

Analysis of retinal isomer composition was carried out using a previously reported method^30^. The retinal oxime extracted from *Halobacterium salinarum* bacteriorhodopsin (HsBR) in the purple membrane (PM) was used as a reference. For measurements under dark conditions, *Psu*ACR1 and HsBR samples were kept in the dark for 1 week at 4 °C. For measurements under light conditions, the samples were respectively illuminated with green (530 nm) and orange (590 nm) LED light for 5 min before retinal oxime extraction. The concentrations of retinal oximes were calculated from peak areas of HPLC chromatograms.

### Spectroscopic measurements

UV-visible absorption spectra were measured at 25 °C using a UV-1800 spectrophotometer (Shimadzu Corp., Kyoto, Japan). The protein concentration was adjusted to an optical density at 535 nm of 0.5–0.6. For the analysis of Cl^−^ concentration dependent spectral changes, the Hill equation was used to determine the Cl^−^ binding affinity in the initial state as follows:

$${\lambda }_{max}=A+B\times \frac{{[C{l}^{-}]}^{n}}{{{K}_{d}}^{n}+{[C{l}^{-}]}^{n}}$$

where *A*, *B*, [Cl^−^], *K*_d_, and *n* represent the offset, the amplitude of λ_max_ change, the Cl^−^ concentration, the dissociation constant, and Hill coefficient, respectively.

Flash-photolysis experiments were carried out at 20 °C using a homemade system as reported previously^22,24^. Data for time-dependent absorption changes from 380 nm to 700 nm every 10 nm were obtained. The number of data acquisitions was 30 for each wavelength. Data were analyzed by the sequential model as reported previously^28,29^;

$${P}_{0}\to {P}_{1}\to {P}_{2}\to {P}_{3}\to {P}_{4}\to {P}_{0}$$

where *P*_0_ and *P*_1_–*P*_4_ represent the initial state and the 1^st^–4^th^ kinetically defined states, respectively. All data for the time-dependent absorption changes were simultaneously fitted with a sum of 4 exponential decay functions in this study. The number of exponents was determined by the reductions in the standard deviation of the residuals (Supplementary Fig. S1). In the *P*_1_–*P*_4_ states, physically defined photo-intermediates such as K, L, and M were populated at equilibrium. Details for the analysis are described in our previous reports^25,26,29^.

## Electronic supplementary material

Supplementary Information
