# Supplementary material for: Implications for the impairment of the rapid channel closing of Proteomonas sulcata anion channelrhodopsin 1 at high Cl− concentrations
Source: Sci Rep. 2018 Sep 7;8:13445. doi: 10.1038/s41598-018-31742-6 (PMC6128917; doi:10.1038/s41598-018-31742-6)
Supplement: Supplementary file 1 — Supplementary Information [file 41598_2018_31742_MOESM1_ESM.docx]

**Supplementary Information**

**Implications for the impairment of the rapid channel closing of *Proteomonas sulcata* anion channelrhodopsin 1 at high Cl^-^ concentrations**

Takashi Tsukamoto, Chihiro Kikuchi, Hiromu Suzuki, Tomoyasu Aizawa,

Takashi Kikukawa & Makoto Demura

Contents:

**Supplementary Figure S1.** Number of exponents for global fitting analysis.

**Supplementary Figure S2.** Flash-photolysis data of *Psu*ACR1 in the presence of

4000 mM Cl^-^.

**Supplementary Figure S3.** Amino acid mapping on the crystal structure of *Cr*ChR2

and absorption spectra of *Psu*ACR1-A93E.

**Reference**

**Supplementary Figure S1.**


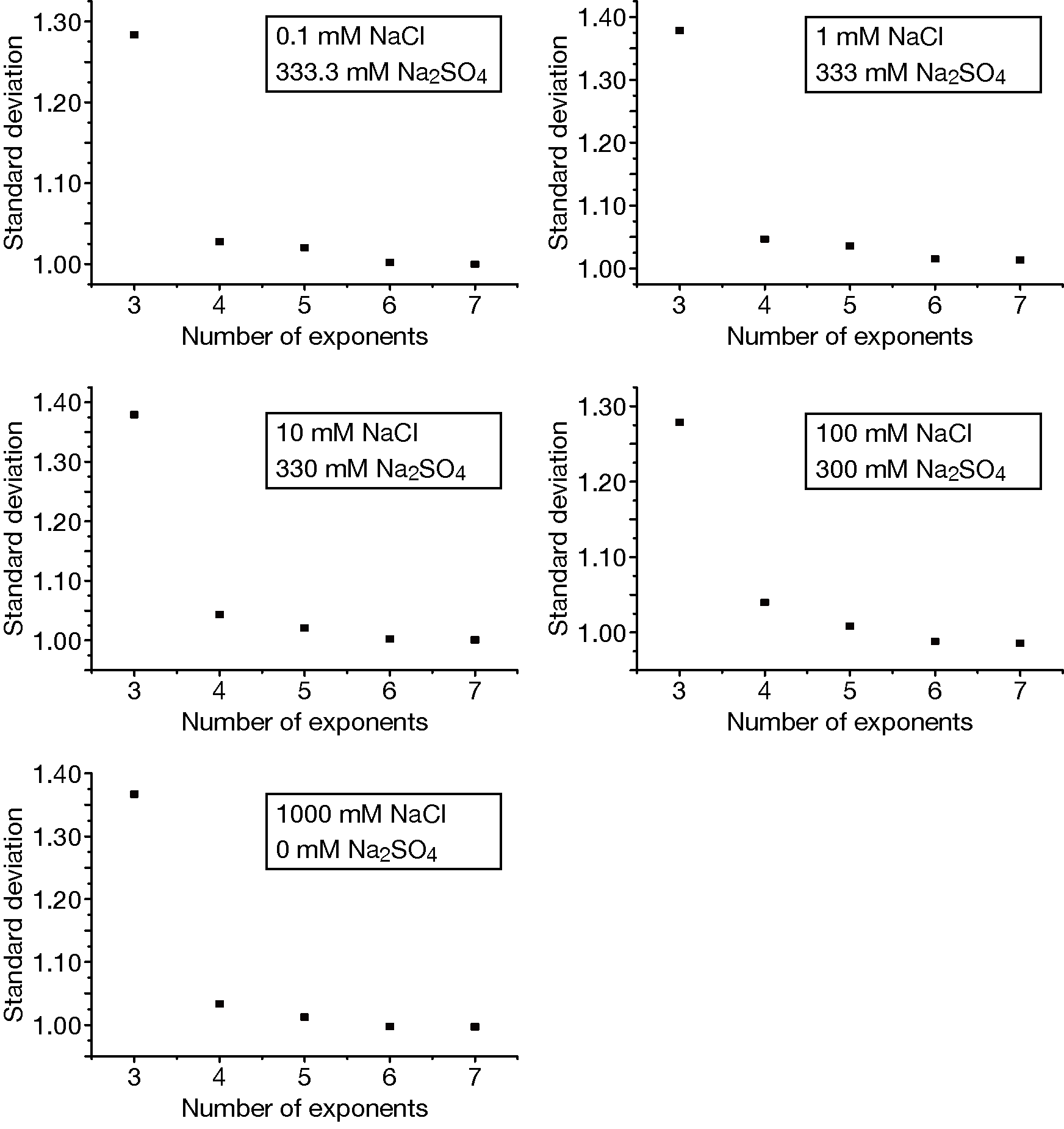


**Supplementary Figure S1.** Number of exponents for global fitting analysis in this study. The appropriate number of exponents was determined as 4 from the reductions in the standard deviation of the residuals.

**Supplementary Figure S2.**


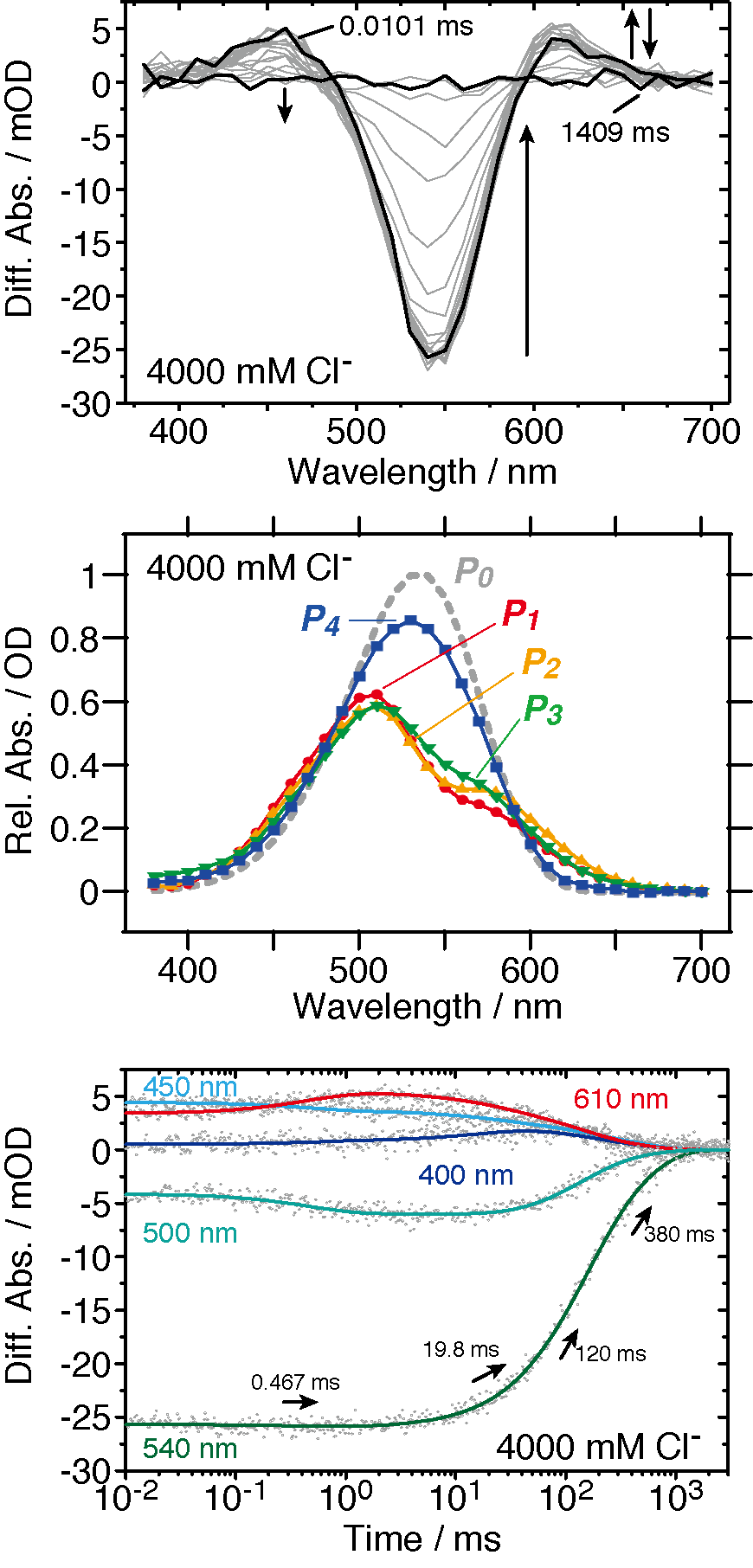


**Supplementary Figure S2.** Flash-photolysis data of *Psu*ACR1 in the presence of 4,000 mM NaCl (0 mM Na_2_SO_4_). (Upper) Flash-induced light minus dark difference spectra. (Middle) Absorption spectra of the kinetically defined states *P_1_* – *P_4_*. *P_0_* spectrum as noted; the grey broken line represents pure retinal spectrum. (Lower) Time evolution of the flash-induced absorption changes (grey dots) of the initial state (540 nm), K and P_600_ (610 nm), L (500 nm), P_450_ (450 nm), and M (400 nm), respectively. Fitting curves are shown in solid lines with colors. Time constants τ_1_ – τ_4_ are shown in the same panel.

**Supplementary Figure S3**

**
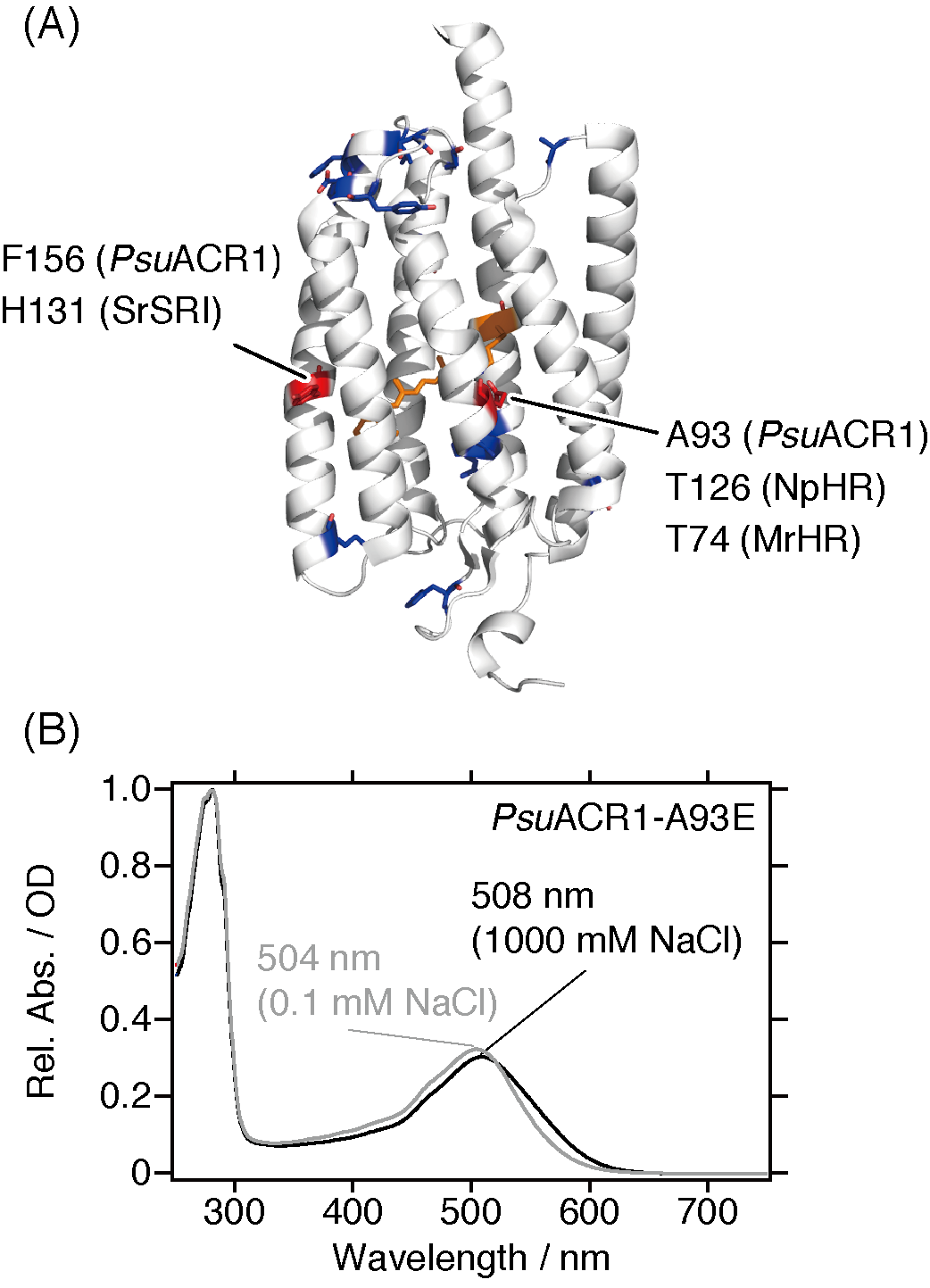
**

**Supporting Figure S3.** Candidate amino acids involved in the initial Cl^-^ binding. (**A**) Amino acids of *Psu*ACR1 are mapped on the crystal structure of *Cr*ChR2 (PDB ID: 6EID) ^1^. Amino acids shown in red are Ala93 and Phe156 in *Psu*ACR1. Corresponding residues in NpHR (Thr126), MrHR (Thr74) and SrSRI (His131) are also indicated. Amino acids shown in blue are positively charged residues, including Arg, Lys and His. (**B**) Absorption spectra of *Psu*ACR1-A93E in the presence of 0.1 mM (333.3 mM Na_2_SO_4_, grey) and 1,000 mM NaCl (0 mM Na_2_SO_4_, black).

**Reference**

1. Volkov, O. *et al.* Structural insights into ion conduction by channelrhodopsin 2. *Science* **358,** eaan8862 (2017).
